# Tetraploid Embryonic Stem Cells Maintain Pluripotency and Differentiation Potency into Three Germ Layers

**DOI:** 10.1371/journal.pone.0130585

**Published:** 2015-06-19

**Authors:** Hiroyuki Imai, Kiyoshi Kano, Wataru Fujii, Ken Takasawa, Shoichi Wakitani, Masato Hiyama, Koichiro Nishino, Ken Takeshi Kusakabe, Yasuo Kiso

**Affiliations:** 1 Laboratory of Veterinary Anatomy, Joint-Faculty of Veterinary Medicine, Yamaguchi University, Yamaguchi, Japan; 2 Laboratory of Developmental Biology, Joint-Faculty of Veterinary Medicine, Yamaguchi University, Yamaguchi, Japan; 3 Biomedical Science Center for Translational Research (BSCTR), The United Graduate School of Veterinary Science, Yamaguchi University, Yamaguchi, Japan; 4 Laboratory of Applied Genetics, Graduate School of Agricultural and Life Science, The University of Tokyo, Tokyo, Japan; 5 Laboratory of Veterinary Biochemistry and Molecular Biology, Miyazaki University, Miyazaki, Japan; Rutgers University -New Jersey Medical School, UNITED STATES

## Abstract

Polyploid amphibians and fishes occur naturally in nature, while polyploid mammals do not. For example, tetraploid mouse embryos normally develop into blastocysts, but exhibit abnormalities and die soon after implantation. Thus, polyploidization is thought to be harmful during early mammalian development. However, the mechanisms through which polyploidization disrupts development are still poorly understood. In this study, we aimed to elucidate how genome duplication affects early mammalian development. To this end, we established tetraploid embryonic stem cells (TESCs) produced from the inner cell masses of tetraploid blastocysts using electrofusion of two-cell embryos in mice and studied the developmental potential of TESCs. We demonstrated that TESCs possessed essential pluripotency and differentiation potency to form teratomas, which differentiated into the three germ layers, including diploid embryonic stem cells. TESCs also contributed to the inner cell masses in aggregated chimeric blastocysts, despite the observation that tetraploid embryos fail in normal development soon after implantation in mice. In TESCs, stability after several passages, colony morphology, and alkaline phosphatase activity were similar to those of diploid ESCs. TESCs also exhibited sufficient expression and localization of pluripotent markers and retained the normal epigenetic status of relevant reprogramming factors. TESCs proliferated at a slower rate than ESCs, indicating that the difference in genomic dosage was responsible for the different growth rates. Thus, our findings suggested that mouse ESCs maintained intrinsic pluripotency and differentiation potential despite tetraploidization, providing insights into our understanding of developmental elimination in polyploid mammals.

## Introduction

In plants and nonmammalian animals, polyploidization has conferred some survival advantages, such as tolerance to genome damage, the requirement for fewer cells in organs, and flexibility and strength of tissues [1]. Whole genome duplication of diploidy, yielding polyploidy, has been utilized for evolution and species differentiation, resulting in dramatic evolutional changes, such as the emergence of teleost fishes [2]. Despite the prevalence of polyploidy in amphibians and fishes, polyploid animals are often sterile [3–5]. In general, polyploidization is an infrequent phenomenon in vertebrates; indeed, polyploidy does not occur naturally in mammals. Spontaneous duplication of the mammalian genome happens in less than 1% of fertilizations, e.g., 0.1% in mice, 0.4% in rats, 0.3% in rabbits, and 0.1–3.4% in pigs [6, 7].

Tetraploid mouse embryos can be produced by artificial methods such as electrofusion of two-cell-stage embryos by electrical stimulation [8–10]. This electrofusion method shows high efficiency and stably produces embryos consisting entirely of tetraploid cells in the organs of mice [11, 12]. After electrofusion, tetraploid embryos can be developed to the blastocyst stage in standard embryo culture medium *in vitro*, which implies that tetraploid cells have normal cellular functions in the early embryo before implantation [13–15].

After implantation, mouse polyploid embryos exhibit abnormalities in the brain, heart, eye, gonads, and vertebral bones [16–18], which consequently cause abortion at various developmental stages. Mouse tetraploid embryos have been reported to develop to midgestation, but abort spontaneously *in vivo* [12, 19–21], suggesting that a unique mechanism for rejection of acute alterations in the original genome dosage exists during mammalian embryogenesis. However, the details of such a mechanism are not fully understood.

In blastocysts, cell lineage into the embryonic or extra-embryonic tissue has been already determined [22]. Embryonic stem cells (ESCs) are established from the inner cell mass (ICM), which differentiates into various embryonic tissues. ESCs sustain the pluripotency to be differentiated and can be induced to differentiate into all types of tissues, including germ cells, under specific culture conditions [23–25]. Analysis of the properties of ESCs may facilitate a better understanding of early development potential as a derivation from the original embryo because the gene expression profiles of ESCs are similar to those of the ICMs of blastocysts.

The purpose of this study was to examine whether changes in the genome dosage affected early mammalian development using artificially tetraploidized ESCs to further determine the cause of the developmental elimination of polyploid mammals. Our results showed that polyploidization may not interfere with the intrinsic pluripotency of ESCs.

## Materials and Methods

### Experimental animals

All mice used in this study were bred at Yamaguchi University. All procedures were approved by the experimental Animal Care and Use Committee of Yamaguchi University (protocol number: 180). The mice were housed in groups of 2–4 with white pine shavings as bedding under a 12-h:12-h photoperiod (lights on at 07:00), with *ad libitum* access to water and food. All mice were purchased from Charles River Laboratories Japan, Inc. (Yokohama, Japan).

### Production of tetraploid embryos

BDF1 female mice (n = 30) were superovulated at 8 weeks of age by intraperitoneal (i.p.) injection of 7.5 IU pregnant mare’s serum gonadotropin (PMSG; Serotropin, Aska Pharmaceutical Co., Ltd., Tokyo, Japan) and 7.5 IU human chorionic gonadotropin (hCG; Gonatropin 3000, Aska Pharmaceutical Co., Ltd.) with a 48-h interval for generation of tetraploid embryonic stem cells (TESCs). Following hCG injection, the females were mated with B6C3F1 males; 12:00 on the day when a vaginal plug was first detected was counted as 0.5 days postconception (d.p.c.). Two-cell-stage embryos were collected at 1.5 d.p.c. by flushing of the oviducts in M2 medium (Sigma, St. Louis, MO, USA). Two-cell embryos were washed with an electrofusion buffer (0.3 M d-mannitol [Sigma], 0.3% bovine serum albumin [BSA; Sigma], and 0.1% polyvinylpyrrolidone) and transferred to a gold electrode chamber attached to an LF301-OS (BEX Co., Ltd., Tokyo, Japan). Two-cell embryos were electrified with 5 V for 5 s to align the embryos parallel to the electrode and with 100 V for 40 ms to electrofuse the embryos. After 30 min, the fused embryos were selected, washed in M2 medium, and cultured to the blastocyst stage in M16 medium (Sigma) in a humidified atmosphere of 5% CO_2_ in air at 37°C.

### Establishment of TESCs

After removal of the zona pellucida by acidic Tyrode’s solution (Sigma), diploid or tetraploid embryos were placed on a gelatin-coated tissue culture dish with mitomycin-C-treated mouse embryonic fibroblast (MEF) feeder cells and cultured in ESGRO Complete plus serum-free grade medium (Merck Millipore, MA, USA) supplemented with 25% Knockout SR (Life Technologies Japan Corporation, Tokyo, Japan), 50 U penicillin, and 50 μg/mL streptomycin. After 4–5 days, the colonies were collected using a glass capillary, trypsinized by PET solution (0.25% trypsin-EDTA), transferred to a new gelatin-coated cell culture dish with mitomycin-C-treated MEFs, and cultured in fresh medium; these cells were designated as P1. Typical round-shaped colonies as ESCs were picked up, cryopreserved using cell freezing medium (DMSO Hybri-Max, Sigma), and stored in liquid nitrogen. Established diploid ESCs or tetraploid ESCs (TESCs) were cultured on gelatin-coated 35-mm cell culture dishes with mitomycin-C-treated MEFs in fresh medium until confluent. The cells were then trypsinized, and the cell number was adjusted for passaging. We also established TESCs expressing transgenic chicken β-actin-driven enhanced green fluorescent protein (EGFP) by the method described above.

### Karyotyping

ESCs and TESCs were incubated with 0.05 μg/mL colchicine (Wako Pure Chemical Industries, Ltd., Osaka, Japan) for 5 h. After trypsinization with PET solution, the cells were resuspended in hypotonic solution (1% sodium citrate) at room temperature (RT) for 20 min, fixed in Carnoy's solution for 30 min, and dropped onto glass slides. After trypsinization with phosphate-buffered saline (PBS) for 1 min, the glass slides were washed with fetal bovine serum (FBS)-PBS solution. Chromosomes were stained with Giemsa stain solution. Eight metaphase spreads were analyzed.

### Flow cytometric analysis

ESCs and TESCs were incubated with 0.05 μg/mL colchicine (Wako) for 5 h. After trypsinization with PET solution, 1 × 10^6^ cells were washed in 600 μL of ice-cold PBS, fixed in 1.4 mL of ice-cold 100% ethanol, and incubated for 1 h at 4°C. After removing the ethanol by centrifugation, the cells were resuspended in 1 mL PBS with 100 μg/mL RNase A and incubated for 1 h at RT. Next, 40 μL propidium iodide solution (40 μg/mL) was added to the cells, and the mixture was incubated for 5 min at RT and filtered through 40-μm mesh filters (Kyoshin Rikoh Inc., Tokyo, Japan) prior to analysis. Analysis was performed on a BD Accuri C6 Flow Cytometer (BD Becton, Dickinson and Company, NJ, USA).

### Cell proliferation analysis

To examine growth curves, ESCs and TESCs were plated at 2 × 10^4^ cells/well in 24-well microplates (AGC Techno Glass Co., Ltd., Shizuoka, Japan) and cultured in culture medium. At appropriate times, the cells were rinsed with PBS, treated with trypsin, and counted with a TC20 Automated Cell Counter (Bio-Rad Laboratories, CA, USA) for 1.5 consecutive days.

### Alkaline phosphatase staining (APS)

After fixation with Lillie buffer solution, APS was performed using an Alkaline Phosphatase Detection Kit (SCR004; Alkaline Phosphatase Detection Kit, Merck Millipore).

### Embryoid body (EB) formation

After trypsinization with PET solution, ESCs and TESCs were plated at 2 × 10^4^ cells/well in PrimeSurface 96U plates (MS-9096U, Sumitomo Bakelite Co., Ltd., Tokyo, Japan) and cultured in DMEM medium (Life Technologies Japan Corp.) supplemented with 10% FBS (Biological Industries Israel Beit-Haemek Ltd., Kibbutz Beit-Haemek, Israel), 10% Knockout SR, 1% MEN non-essential amino acid solution (100×; Sigma), 1% 2-mercaptoethanol (Merck Millipore), 50 U penicillin, and 50 μg/mL streptomycin for 10 days. The EBs were cultured for 10 days, and total RNA was then extracted.

### Teratoma formation

After trypsinization with PET solution, approximately 1–2 × 10^6^ ESCs or TESCs were resuspended in PBS with BD Matrigel hESC-qualified Matrix (BD Becton, Dickinson and Company) and subcutaneously injected into 8-week-old female immune-deficient nude mice (BALB/c-nu/nu) and SCID mice (C. B-17/Icr-Prkdc<scid>/CrlCrlj) to test for teratoma formation. We randomly allocated the animals to the experimental group. After 4–8 weeks, the mice were sacrificed by cervical dislocation, and the teratomas were excised, fixed in Lillie buffer solution, embedded in paraffin, sectioned, and stained with hematoxylin-eosin for histochemical analysis.

### Quantitative real-time reverse transcription polymerase chain reaction (qRT-PCR)

For qRT-PCR, total RNA was isolated from ESCs, TESCs, EBs, and MEFs using the ReliaPrep RNA Cell MiniPrep System (Promega, Madison, WI, USA). The total RNA of each sample was quantitatively measured using a spectrophotometer (ND-1000, NanoDrop, Thermo Fisher Scientific Inc., MA, USA), and cDNA was synthesized from 1 μg of total RNA with a QuantiTect Reverse Transcription Kit (Qiagen K. K., Tokyo, Japan). Table 1 lists the primers used to detect *Gapdh*, *Nanog*, *Oct3/4*, *Sox2*, *Nestin*, *Sox1*, *Brachyury*, *Mixl1*, *Acvr1*, *Sox17*, *Hand1*, and *Elf5*. qRT-PCR was performed using an Applied Biosystems StepOnePlus Real Time PCR System (Life Technologies Japan Corporation) in a 20-μL volume with 1 μM each of the forward and reverse primers and Power SYBR Green PCR Master Mix (Applied Biosystems). The amplification protocol consisted of 95°C for 10 min followed by 40 cycles at 95°C for 15 s and 60°C for 60 s. Relative gene expression was determined by normalization to the internal standard gene, *Gapdh*.

**Table 1:** Primer Sequences.

*Gene*	Forward Primer (5' → 3')	Reverse Primer (5' → 3')
***Gapdh***	GTGCTGAGTATGTCGTGGAGTC	CATACTTGGCAGGTTTCTCCAG
***Nanog***	TCCTTGCCAGGAAGCAGAAGATGC	CACTGGTTTTTCTGCCACCGCTTG
***Oct3/4***	GCATACGAGTTCTGCGGAGGGATG	GGACTCCTCGGGAGTTGGTTCCAC
***Sox2***	GCACATGAAGGAGCACCCGGATTA	TGCATCATGCTGTAGCTGCCGTTG
***Nestin***	AGGCGCTGGAACAGAGATTGGAAG	TCCAGGTGTCTGCAAGCGAGAGTT
***Sox1***	GCCAAGACAGCGTGCCTTTGATTT	CAATCTGCATCCCGGTTCACACAG
***Brachyury***	CATCGGAACAGCTCTCCAACCTAT	GGTACCATTGCTCACAGACCAGAG
***Mixl1***	CGAGTCCAGGATCCAGGTGTGGTT	ATCCGGAACGTGGTTCACATCTGC
***Acvr1***	GAGAAGTCATGGTTCAGGGAGAC	GCAGCTAACCGTATCCAGAGTAGT
***Sox17***	CAGCATATGCAGGACCACCCCAAC	CAGTCCCTGGCAGTCCCGATAGTG
***Hand1***	CTGCGCCTGGCTACCAGTTACAT	GTGCGCCCTTTAATCCTCTTCTCG
***Elf5***	CTGGGAATGGCTCCAATTCTGCTG	ACCTTGCGAGCGAATGTTCTGGAG

### Quantitative combined bisulfite restriction analysis (COBRA)

To confirm the DNA methylation state, bisulfite PCR-mediated restriction mapping (known as the COBRA method) was performed. Sodium bisulfite treatment of genomic DNA was carried out using an EZ DNA Methylation-Gold kit (Zymo Research Corporation, CA, USA). PCR amplification was performed using BIOTAQ HS DNA polymerase (Bioline Ltd., London, UK) with specific primers for *Oct3/4* and *Nanog* (Table 2). After digestion with restriction enzymes, HpyCH4IV quantitative-COBRA coupled with a Shimadzu MCE-202 MultiNA Microchip Electrophoresis System (Shimadzu Corporation, Kyoto, Japan) was carried out for quantification of the DNA methylation level.

**Table 2:** Primer sequence in COBRA.

Gene	Forward Primer (5' → 3')	Reverse Primer (5' → 3')	Product size	Enzyme	Digestion pattern
***Oct3/4***	GCATACGAGTTCTGCGGAGGGATG	GGACTCCTCGGGAGTTGGTTCCAC	473	HpyCH4IV	278, 195
***Nanog***	GCACATGAAGGAGCACCCGGATTA	TGCATCATGCTGTAGCTGCCGTTG	258	HpyCH4IV	192, 66

### Generation of chimeric embryos

Two-cell-stage ICR embryos were collected at 1.5 d.p.c. by flushing of the oviducts in M2 medium (Sigma). The embryos were cultured to the morula stage in M16 medium (Sigma), and the zona pellucida was then removed by acidic Tyrode’s solution at the morula stage. One diploid morula-stage embryo and dispersed TESCs (10–15 cells) were aggregated in the bottom of a culture dish in a well created with a needle (DN-9, Biological Laboratory Equipment Maintenance and Service Ltd., Budapest, Hungary). The aggregated embryos were washed and cultured to the blastocyst stage in M16 medium (Sigma) in a humidified atmosphere of 5% CO_2_ in air at 37°C.

### Immunocytochemistry

ESCs and TESCs were cultured with mitomycin-C-treated MEFs on a cover slip. After 48 h of culture, the cells were fixed with 0.1% PVP and 3.5% paraformaldehyde at 4°C for 30 min. After permeabilization by 0.2% Triton-X 100 in PBS for 20 min and blocking with 2% FBS in PBS for 15 min, the cells were incubated with primary antibodies shown in S1 Table at 4°C overnight, then with fluorescein isothiocyanate (FITC)- or rhodamine-conjugated secondary antibodies at room temperature for 45 min. After washing with PBS, the cover slips were mounted on glass slides with Vectashield mounting medium (H-1000, Vector Laboratories Inc., Burlingame, CA, USA), and the cells were then observed using a confocal laser scanning microscope (LSM 700, Olympus Corporation, Tokyo, Japan) and analyzed using the accessory software (ZEN, Carl Zeiss Microscopy Co., Ltd., Tokyo, Japan).

### Statistical analysis

Mann-Whitney tests were used to detect significant differences between the experimental groups. Differences were considered significant at *P* < 0.05 (StatView software, SAS Institute, NC, USA).

## Results

### Establishment and characterization of tetraploid ESCs from murine tetraploid blastocysts

**Fig 1:**
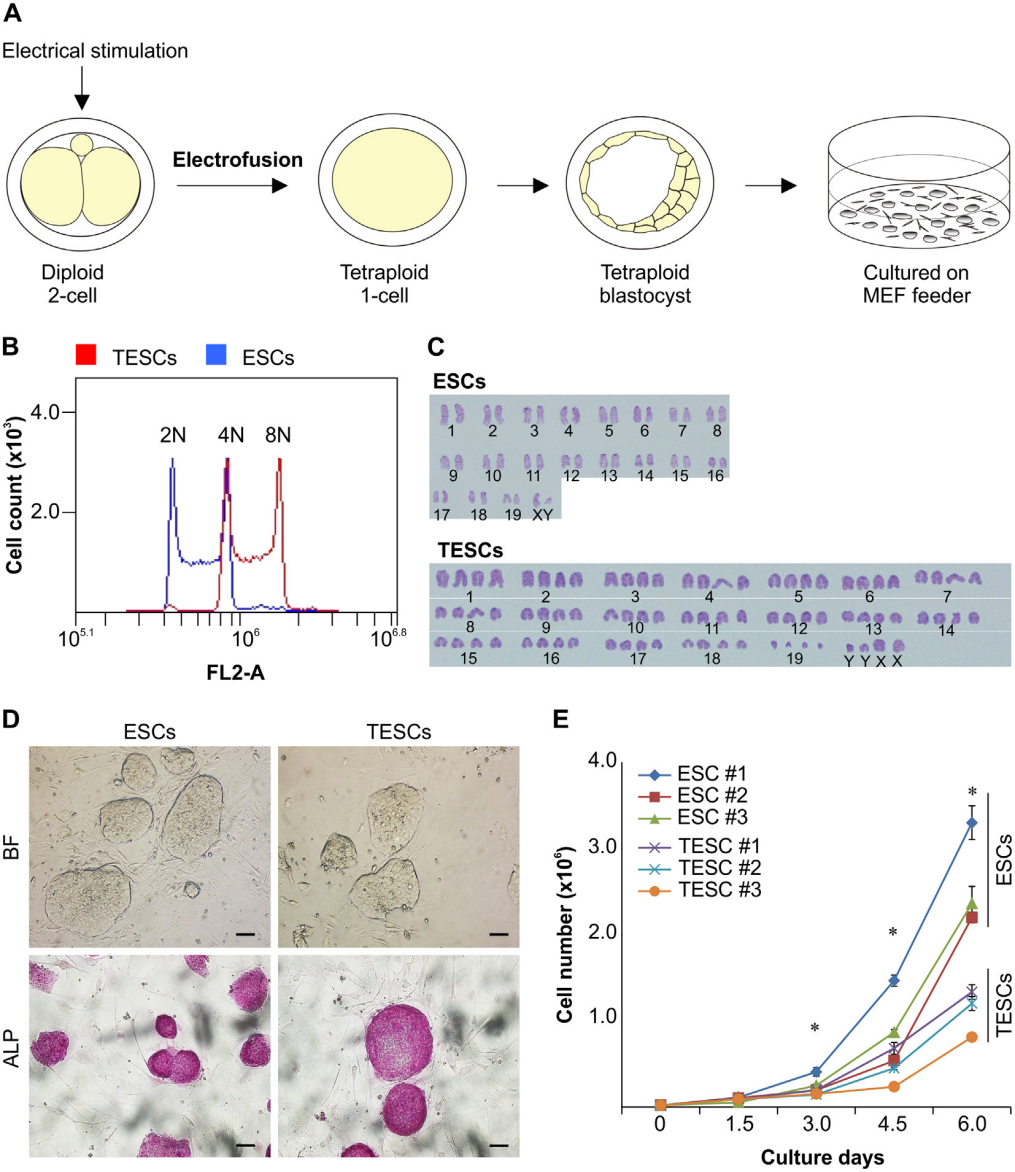
Production and Characterization of Tetraploid Embryonic Stem Cells (TESCs). (**A**) Diagram of production of TESCs with elecrofused diploid embryos. Two or three embryos were seeded onto a mouse embryonic fibroblast (MEF) feeder layer. (**B**) Flow cytometry analysis of DNA content after 10 passages. A representative figure is shown. (**C**) Karyotyping analysis of metaphase chromosome spreads. Tetraploid ESCs normally had 80 chromosomes, while control diploid ESCs had 40 chromosomes at passage 10. A representative figure is shown. (**D**) Proliferating TESCs formed typical round-shaped mouse ESC colonies with clear boundaries similar to control ESCs. TESC colonies also stained positive for the control ESC-positive marker alkaline phosphatase (ALP). A representative figure is shown. (**E**) Growth rates. The relative cell number was based on the cell number at day 0 (2 × 10^4^ cells). All data represent the mean and SEM (n = 3). *P < 0.001. Scale bar, 100 μm.

We first generated tetraploid mouse embryos by electrofusion of two-cell embryos of BDF1 male × BDF1 superovulated female matings and cultured these embryos to blastocyst embryos. We generated several ESC lines by culturing either the diploid or tetraploid blastocysts on MEFs after removal of the zona pellucida (Fig 1A). In total, nine ESC lines were established from the tetraploid blastocysts (establishment rate, 15%). On the other hand, 15 ESC lines were established from diploid blastocysts (88%; S2 Table). In this study, we used three lines from each cell type (i.e., diploid ESCs, ESCs #1, #2, and #3; and tetraploid ESCs, TESCs #1, #2, and #3). After several passages, we confirmed the DNA content by flow cytometric analysis (Fig 1B and S1 Fig). The TESC lines generated in this study had twice as much DNA as control ESC lines at passage 2–8 (Fig 1B and S1 Fig). TESCs normally had 80 chromosomes, while diploid ESCs had 40 chromosomes at passage 8, as shown by karyotyping and chromosome spreads (Fig 1C and S1 Fig). TESC lines proliferated in culture for at least 25 passages and formed typical round-shaped mouse ESC colonies with clear boundaries, similar to control ESCs (Fig 1D). TESCs also stained positive for alkaline phosphatase (ALP), similar to control ESCs (Fig 1D).

Next, we calculated the relative proliferation rates of the cells at each period in the three lines of TESCs and three lines of ESCs based on the cell number at day 0. Despite similarities in morphology and the expression of the ESC-positive marker ALP, the relative proliferation rate of TESCs was significantly lower than that of control ESCs after 3 days of culture (Fig 1E and S1 Fig); cellular apoptosis and necrosis did not account for this difference (S1 Fig). These results indicated that TESC lines were successfully established using an orthodox method and showed that TESCs exhibited similarities in shape, ALP staining, and genome dosage stability, but differences in the proliferation rate compared to ESCs.

### Expression of ESC markers

**Fig 2:**
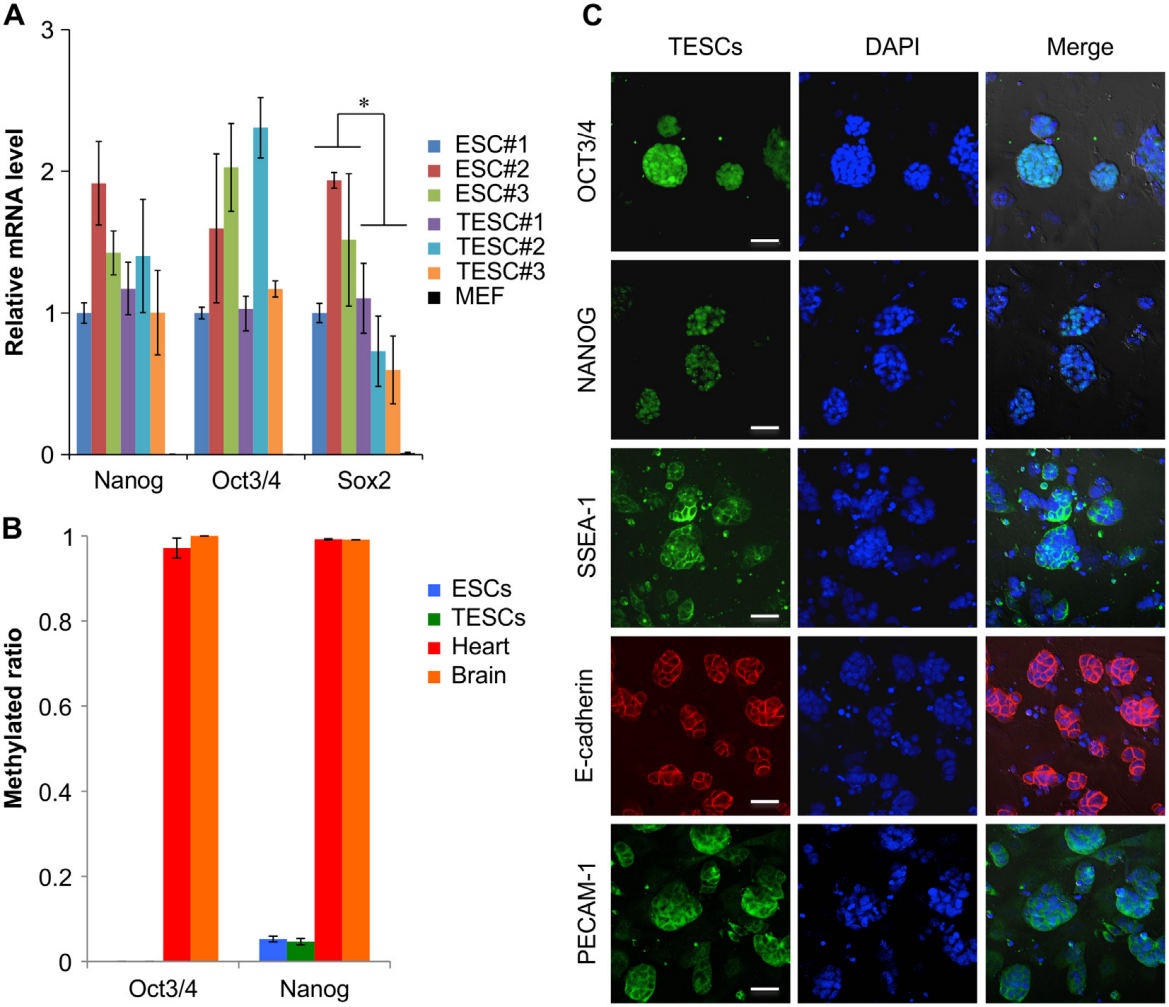
Expression and Epigenetic Analysis of TESCs. (**A**) Relative expression of pluripotency markers by quantitative real-time RT-PCR analysis at passage 10. All data represent the mean and SEM (n = 3). *P < 0.05. (**B**) Analysis of DNA methylation profiles. DNA methylation levels in *Oct3/4* and *Nanog* genes were determined by Bio-COBRA in TESCs and ESCs. All data represent the mean and SEM (n = 3). *P < 0.05. (**C**) Immunostaining of pluripotency markers. DAPI was used to stain DNA. A representative figure is shown. Scale bar, 50 μm.

To acquire molecular information regarding the key stem cell factors, qRT-PCR analyses were performed in ESCs and TESCs. TESCs expressed typical pluripotency markers, including *Nanog* and *Oct3/4*, at levels similar to those of control ESCs. In contrast, the relative ratio of *Sox2* mRNA was significantly lower in TESCs than in ESCs. These pluripotency markers were silenced in MEFs as negative controls (Fig 2A: passage 10 and S2 Fig: passage 20). To study the methylation profiles of the promoter region of *Oct3/4* and *Nanog* genes, which are specifically expressed in ESCs, we performed quantitative COBRA (Fig 2B). In the promoter regions for these genes, methylation was low in TESCs, similar to that observed in ESCs, indicating that TESCs retained the normal epigenetic status for these significant reprogramming factors (Fig 2B). Indirect immunofluorescence for NANOG, OCT3/4, and SSEA-1 at passage 5 indicated that all of these pluripotency molecules were expressed at the protein level in TESC colonies, similar to their expression in control ESC colonies (Fig 2C). We also performed indirect immunofluorescence for E-Cadherin and platelet endothelial cell adhesion molecule 1 (PECAM-1), which are both localized at ESC niches. The results indicated that these proteins were also expressed in TESC colonies, similar to their expression in control ESC colonies (Fig 2C and S3 Fig). These data indicated that TESCs expressed key stem cell factors specific for ESCs.

### Differentiation potential of TESCs *in vitro* and *in vivo*

**Fig 3:**
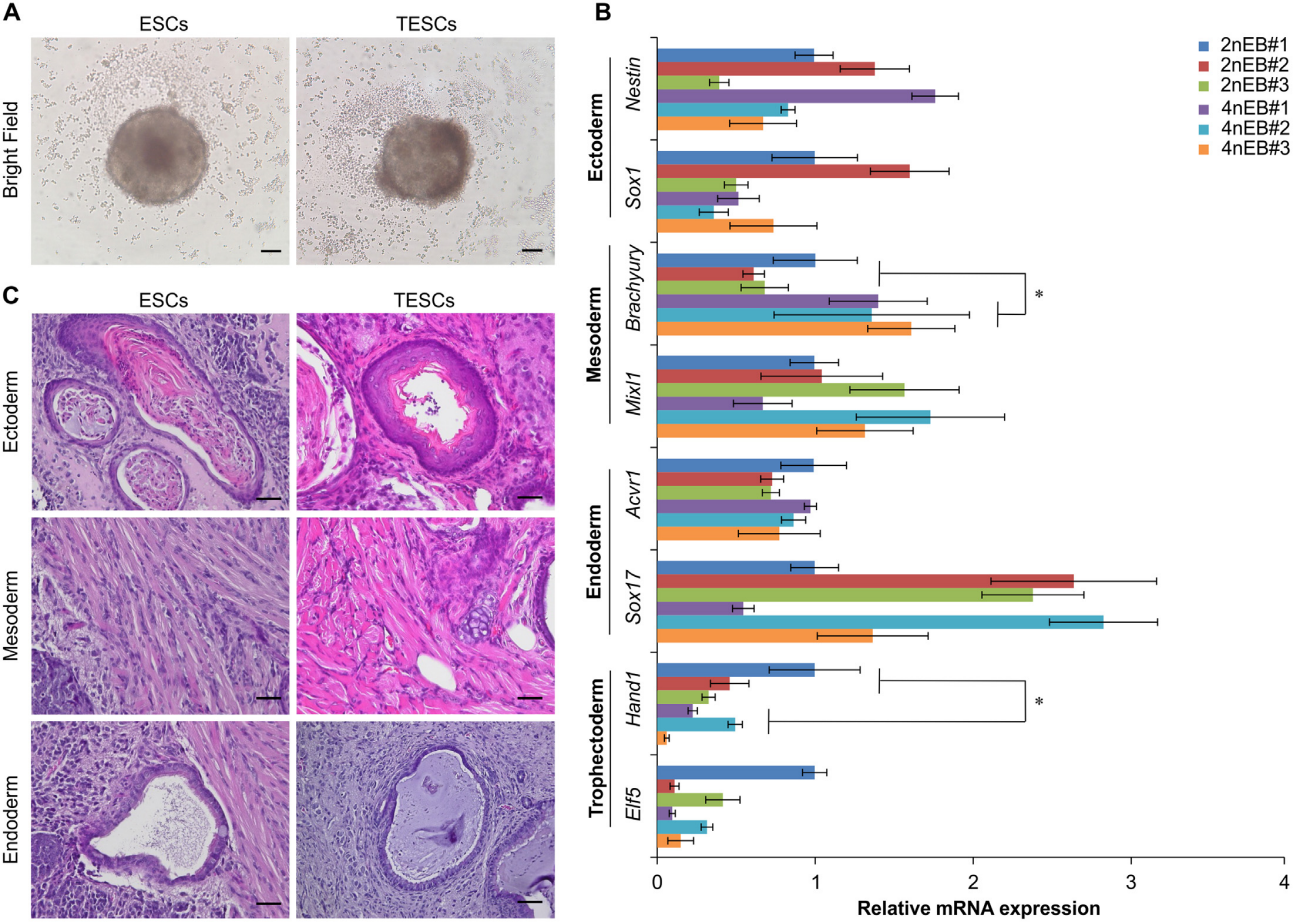
The Differentiation Potential of TESCs. (**A**) Morphology of embryoid bodies generated from TESCs and ESCs. Scale bars, 100 μm. (**B**) Relative expression of various cell lineage marker genes in embryoid bodies generated from TESCs and ESCs by quantitative real-time RT-PCR analysis. All data represent the mean and SEM (n = 3). *P < 0.05. (**C**) Teratoma formation by TESCs. Scale bars, 50 μm.

To investigate the potential of TESCs to differentiate into germ layers cells *in vitro*, we generated EBs by plating TESCs into low-adherence dishes without feeder cells to prevent attachment (Fig 3A). TESCs could successfully form EBs, as judged visually, and the ESC marker genes *Oct4*, *Sox2*, and *Nanog* (data not shown) were downregulated. We further confirmed the expression of markers of the three germ layers by qRT-PCR at 10 days after EB formation (Fig 3B). There were no significant differences in the relative expression levels of ectodermal genes (*Nestin* and *Sox1*) or endodermal genes (*Acvr1* and *Sox17*). A mesodermal gene (*Brachyury*) was significantly upregulated in EBs derived from TESCs compared to those of ESCs, and a trophectodermal gene (*Hand1*) was significantly downregulated in EBs derived from TESCs compared to those of ESCs (Fig 3B).

To investigate the differentiation potential of TESCs *in vivo*, we injected TESCs subcutaneously into immune-deficient nude mice and SCID mice. At 4–8 weeks after injection, teratomas derived from ESCs were formed in a significant proportion of the mice (n = 4, 80%; S3 Table), while the teratomas derived from TESCs were formed in fewer mice (n = 4, 14%; S3 Table). Histological analysis revealed that the teratomas derived from TESCs containing tissues of all three germ layers, including neural tissues, cartilage (ectoderm), muscle (mesoderm), and intestinal epithelia (endoderm; Fig 3C). Our data suggested that TESC-derived cells may have an intrinsic potential to develop into cells of all three germ layers *in vivo*, similar to that of ESC-derived cells. To confirm whether the teratomas derived from TESCs consisted essentially of tetraploid cells, we analyzed the nucleus area of each cell in mesodermal tissues of teratomas. The relative nucleus area of teratoma cells derived from TESCs was significantly larger than that of teratoma cells derived from ESCs (S4 Fig). These results suggested that TESCs could contribute to a wide range of adult tissues.

### Distribution of cells derived from TESCs in embryonic chimeras

**Fig 4:**
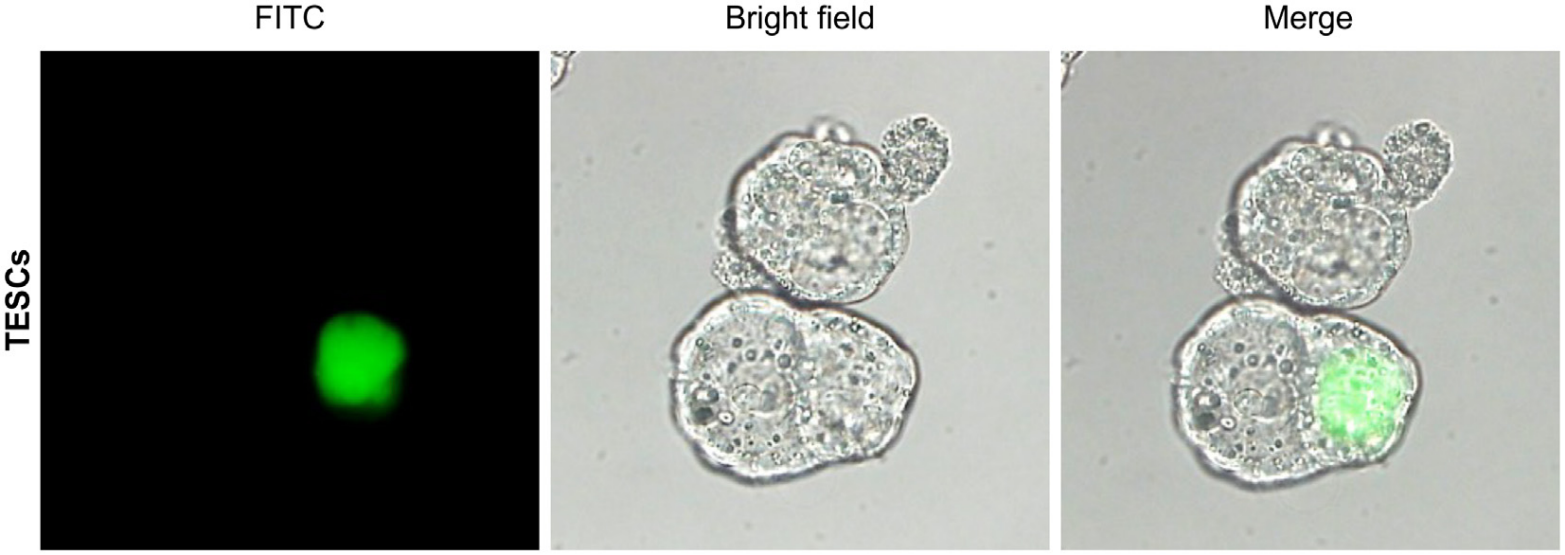
Development of Morula Embryo Aggregates from Injections of *In Vitro* Cultures of EGFP-Expressing TESCs. Morphology, fluorescence, and merged images of aggregated embryos at the blastocyst stage.

Finally, we produced chimeric embryos by aggregating EGFP-positive TESCs with ICR mouse morulas to monitor the developmental contribution of TESCs in early embryos. The chimeric embryos aggregated with dispersed TESCs and a diploid morula embryo to develop normally into a blastocyst stage embryo (Fig 4). Tetraploid cells derived from EGFP-positive TESCs were predominantly located in the ICM of the blastocyst in most of the chimeras (Fig 4). These data indicated that TESCs maintained the ability to participate in ICMs with diploid cells.

## Discussion

In the present study, we produced the several lines of TESCs from tetraploid blastocysts by electrofusion in mice. We found that TESCs maintained intrinsic pluripotency and differentiation potential despite artificial tetraploidization. These results implied that ESCs may have the flexibility to adapt to acute polyploidization in the early development of mammals.

Tetraploid mouse embryos rarely occur in nature, but can be produced by methods that block cell division chemically, such as the use of cytochalasin B [26]. Recently, a new method for transplanting two somatic nuclei into the same enucleated oocyte was developed [27, 28]; however, electrofusion methods are popular and convenient for efficient production of tetraploid embryos in mice. Injection into tetraploid blastocysts is also useful and can result in rapid production of mutant mice derived completely from ESCs [29]. Many studies have shown that mouse tetraploid embryos develop normally until implantation [13–15, 27, 28]. The total number of cells in tetraploid blastocysts is fewer than that in diploid blastocysts [15, 30]. However, the ICM expresses *Oct4* (our unpublished data, [27]), which implies that tetraploid cells of the ICM have the potential to develop into ESCs. We have succeeded in establishing TESCs from mouse tetraploid blastocysts using a 2i culture method [31] to efficiently create ESCs from mouse blastocysts. However, the efficiency of the establishment of TESCs (15%) was quite low compared to that of ESCs (88%). Wen *et al*. distinguished two groups of mouse tetraploid blastocysts based on the presence or absence of the ICM at the blastocyst stage [32]. This low efficiency of establishment of TESCs may reflect the low number of cells composing the ICM [33] and the low numbers of positive cells for *Oct3/4* expression [32, 34].

The first colonies from tetraploid blastocysts were similar to diploid ESCs and emerged at the same time as colonies from diploid blastocysts (our unpublished data). Cells in the pluripotency phase can be defined as naïve or primed. The naïve type has dome-shaped colonies established from pre-implantation embryos as mouse ESCs, while the primed type has flattened colonies established from postimplantation embryos as human ESCs and epiblast stem cells (EpiSCs) [35, 36]. The TESCs established in this study formed typical dome-shaped colonies, suggesting that these TESCs had properties similar to those of mouse ESCs. Our results revealed that the relative growth rate of established TESCs was significantly lower than that of control ESCs during all periods after several passages. Tetraploid embryos likely have a slower cell cycle and consequently fewer cells than their age-matched diploid control embryos [13, 15, 30]. DNA microarray data have shown that genes involved in cell division and the cell cycle, including *Ccnb1* (cyclin B1), are significantly decreased in tetraploid blastocysts compared with diploid blastocysts [15]. TESCs may inherit the cell cycle, cell division, and survivability characteristics of the ICM of the tetraploid blastocyst during *in vitro* stem cell culture environment. In the future, it will be necessary to consider the translational product quantity of cell cycle-related genes and the timing of DNA replication using global gene expression analysis.

The genome dosage of haploid ESCs generated by activation of unfertilized oocytes is unstable; thus, the genome dosage autonomously reverts to the diploid state after several passages or induction of differentiation [37–39]. We confirmed that the genome dosage of TESCs was sustained after more than 20 passages, which indicated that TESCs maintained genetic homogeneity and that cellular polyploidization may be acceptable in the mammalian genome.

TESCs expressed the pluripotent stem cell marker genes *Nanog* and *Oct3/4* at levels similar to those of ESCs; however, *Sox2* expression, which has been used as an embryonic marker for neural cells [40], was significantly lower in TESCs than in ESCs at passage 10. Furthermore, our preliminary analysis of the global gene expression profile revealed that TESCs had a gene expression profile similar to that of ESCs (data not shown). Several markers of early differentiation, including *Cdx2* and *Hand1* (trophectoderm markers) and *Gata4* and *Gata6* (primitive endoderm markers), were silenced in TESCs and ESCs (data not shown). These results implied that the TESCs may exhibit sustained expression of pluripotency genes despite duplication of the genome dosage. Moreover, despite duplication of the genome dosage of TESCs, methylation of the promoter regions of these genes was mostly absent, similar to normal diploid ESCs. The global gene expression patterns and methylation levels of the promoter regions of these genes are similar to those of ESCs produced by transplanting two somatic nuclei into one enucleated oocyte [28]. The viable tetraploid mammal *Tympanoctomys barrerae* (Octodontidae) displays genomic imprinting and X chromosome inactivation, which is partially conserved in the tetraploid genome [41]. Future comprehensive epigenetic analyses are necessary to elucidate the characteristics of TESC pluripotency; however, our local epigenetic analysis and previous studies indicated that similarities in the epigenetic processes between tetraploidy and diploidy may be necessary for the survival of tetraploid mammalian cells.

We also produced chimeric blastocysts between TESCs expressing EGFP and diploid embryos by the aggregation method of injection into morula embryos [42]. In previous studies, tetraploid cells contribute to only extra-embryonic tissues and that ESCs consisted mainly of embryonic tissues in the offspring of these aggregated embryos [27, 43]. Unexpectedly, our results showed that TESCs mainly contributed to the ICM in chimeras with a diploid embryo, implying that the TESCs retained locational potential to remain in the correct location for development of the embryonic lineage and all organs at the blastocyst stage *in vitro*. Interestingly, tetraploid nuclear transfer-derived ESCs do not contribute to implanting embryos [28]. Recently, p53-dependent cell cycle arrest and apoptosis have been shown to have an important role in the degeneration of tetraploid embryos in mice [44]. However, our data revealed that apoptosis and necrosis did not occur in TESCs, indicating that development of tetraploid embryos may be complicated due to interactions between the embryo and uterus. Further studies, including transfer of chimeric embryos into the uterus, are needed to clarify the characteristics of TESC differentiation.

In this study, we also found that teratomas formed following subcutaneous injection of TESCs containing tissues from all three germ layers despite lower efficiency, similar to those formed after injection of ESCs and tetraploid nuclear transfer-derived ESCs [28]. The low efficiency of teratoma formation in TESCs may be associated with cellular competition between donor diploid cells and recipient tetraploid cells in the grafted area. Because of the abnormal development of tetraploid embryos, it is unlikely that teratomas formed from TESCs could develop into normal organs, suggesting that TESCs maintained the potential to develop into all organs *in vivo*, regardless of tetraploidization by electrofusion of two-cell embryos. However, differentiation analyses of the EBs showed differences in the expression of mesodermal (*Brachyury*) and trophectodermal (*Hand1*) genes between ESCs and TESCs. TESCs also exhibited a unique differentiation potential compared with normal ESCs, indicating that tetraploidization did not affect differentiation *in vitro*, but may trigger abnormal development in the mouse tetraploid embryo after implantation *in vivo*. Variations in directional differentiation between TESCs and ESCs may occur. However, further studies are needed to clarify the characteristics of TESC differentiation. Our results showed that TESCs had the potential to differentiate into all germ layers, which are the basic structures of organs in the embryo and were conserved in tetraploid stem cells. Additionally, we did not find any evidence of developmental abnormalities in mouse tetraploid embryos in this study, indicating that elimination of mouse tetraploid embryos may depend on a higher-order organization and coordination during tissue differentiation and/or the interaction between the embryo and placenta during implantation, a unique developmental system in mammals.

## Conclusions

In the present study, we found that TESCs maintained intrinsic pluripotency and differentiation potential, including gene expression profiles and differentiation to germ layers. However, TESCs may be unique in their developmental potential, including directional differentiation. We also found that TESCs had localization potential to the ICM in aggregated chimeras with diploid embryos. These data indicated that ESCs may have substantial flexibility to maintain early development despite tetraploidization using electrofusion of two-cell embryos in mammals. Future studies are required to elucidate the molecular mechanisms of abortion of polyploid mammals *in vivo* for basic developmental biology research due to the unique mechanism for rejection of acute alterations in the original genome dosage in mammals.

## Supporting Information

S1 ARRIVE Checklist ARRIVE guidelines checklist. (PDF)

S1 Fig Analysis of DNA Content in ESCs and TESCs. (**Figure A**) Forward scatter and side scatter in flow cytometry analysis of ESCs (#1) and TESCs (#1). The cells in the R1 area were selected for DNA content analysis. (**Figure B**) Flow cytometry analysis of DNA content of ESCs (#2, #3) and TESCs (#2, #3) after 2–8 passages. (**Figure C**) Metaphase chromosome spreads. Tetraploid ESCs normally had 80 chromosomes, while control diploid ESCs had 40 chromosomes at passage 8. (**Figure D**) Typical round-shaped TESC and ESC colonies on culture days 1.5, 3.0, and 4.5. **(Figure E)** Relative expression of *caspase-3* mRNA by quantitative real-time RT-PCR analysis. All data represent the mean and SEM (n = 3). **(Figure F)** The number of ESCs (#1) and TESCs (#1) negative for trypan blue staining.
(TIF)

S2 Fig Relative Expression of Pluripotency Markers by Quantitative Real-Time RT-PCR Analysis of ESCs (#1) and TESCs (#1) at Passages 5 (p5) and 20 (p20). All data represent the mean and SEM (n = 3).
(TIF)

S3 Fig SSEA-1, E-cadherin, and PECAM-1 Proteins were Expressed in ESCs. DAPI as used to stain DNA. A representative figure is shown. Scale bar, 50 μm.
(TIF)

S4 Fig Sizes of the Nucleus in Cells within Teratomas. The nuclei of cells in ectodermal and mesodermal tissues within teratomas derived from TESCs were approximately twice as large as those from teratomas derived from ESCs.
(TIF)

S1 Table Primary antibodies. (DOCX)

S2 Table Efficiency of establishment of embryonic stem cells from diploid or tetraploid blastocysts. (DOCX)

S3 Table Differentiation of ESCs and TESCs in vivo. (DOCX)
