# Supplementary figures and images for: Tetraploid Embryonic Stem Cells Maintain Pluripotency and Differentiation Potency into Three Germ Layers
Source: PLoS One. 2015 Jun 19;10(6):e0130585. doi: 10.1371/journal.pone.0130585 (PMC4474668; doi:10.1371/journal.pone.0130585)

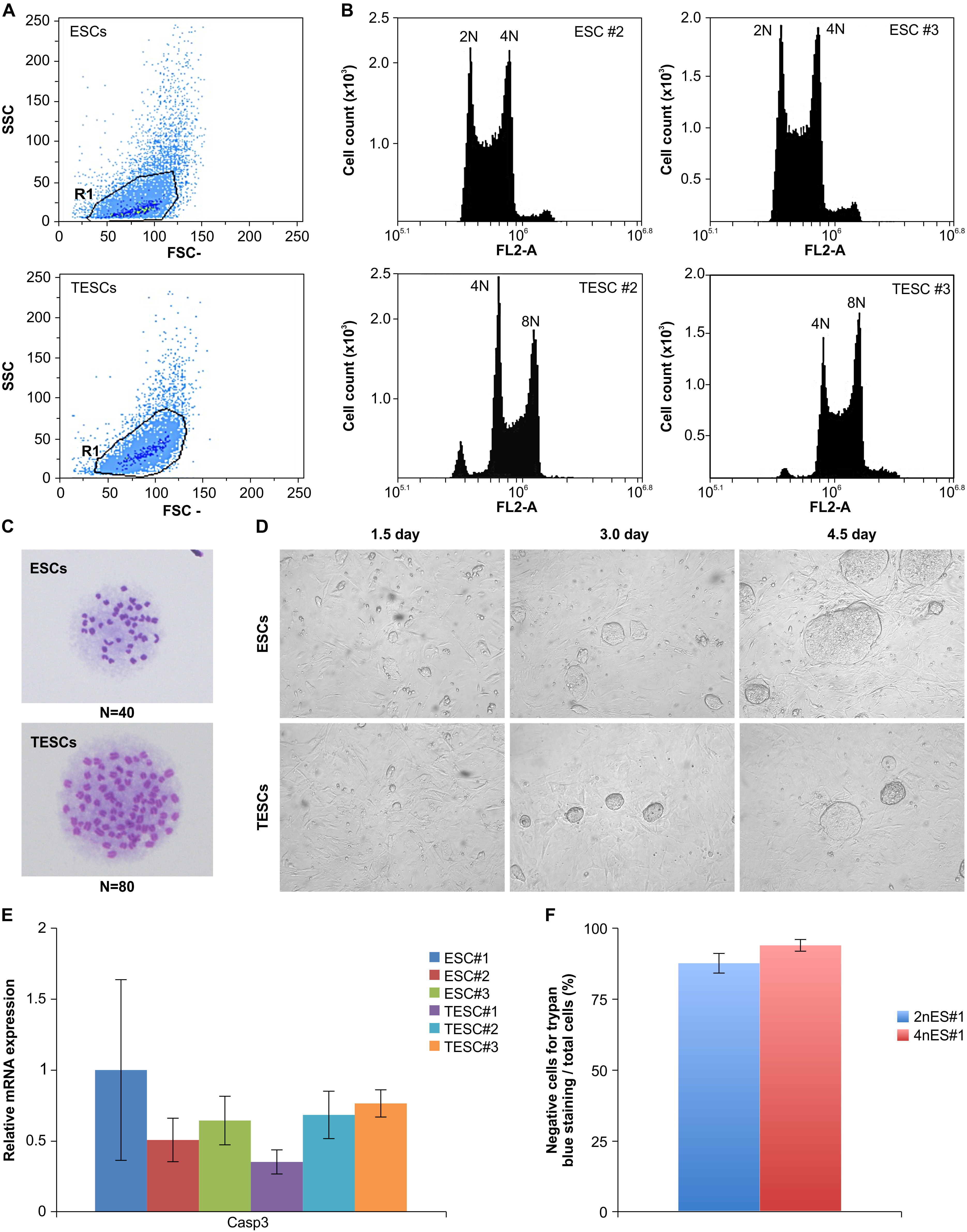

Supplement: S1 Fig — (Figure A) Forward scatter and side scatter in flow cytometry analysis of ESCs (#1) and TESCs (#1). The cells in the R1 area were selected for DNA content analysis. (Figure B) Flow cytometry analysis of DNA content of ESCs (#2, #3) and TESCs (#2, #3) after 2–8 passages. (Figure C) Metaphase chromosome spreads. Tetraploid ESCs normally had 80 chromosomes, while control diploid ESCs had 40 chromosomes at passage 8. (Figure D) Typical round-shaped TESC and ESC colonies on culture days 1.5, 3.0, and 4.5. (Figure E) Relative expression of caspase-3 mRNA by quantitative real-time RT-PCR analysis. All data represent the mean and SEM (n = 3). (Figure F) The number of ESCs (#1) and TESCs (#1) negative for trypan blue staining. (TIF) [file pone.0130585.s002.tif]

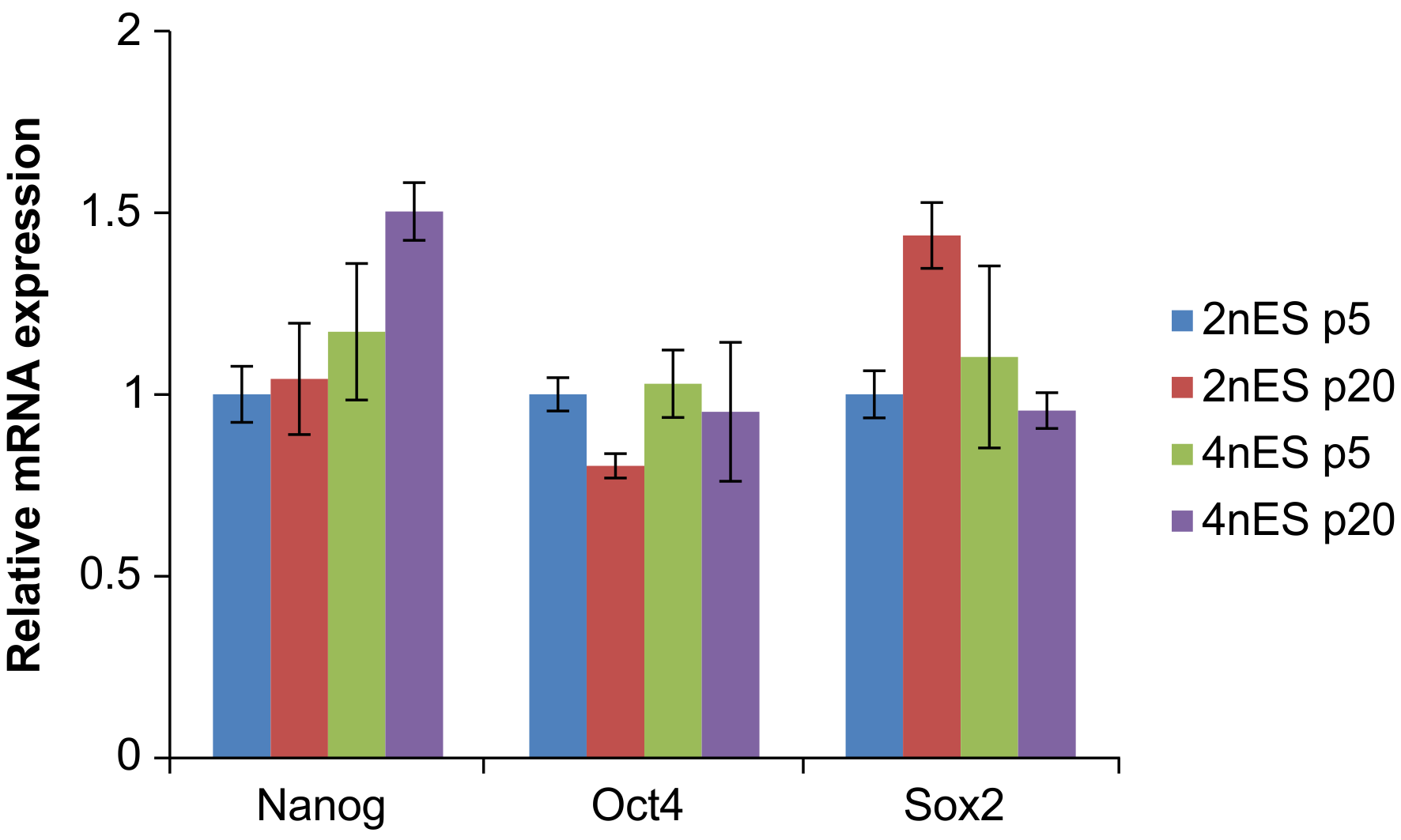

Supplement: S2 Fig — All data represent the mean and SEM (n = 3). (TIF) [file pone.0130585.s003.tif]

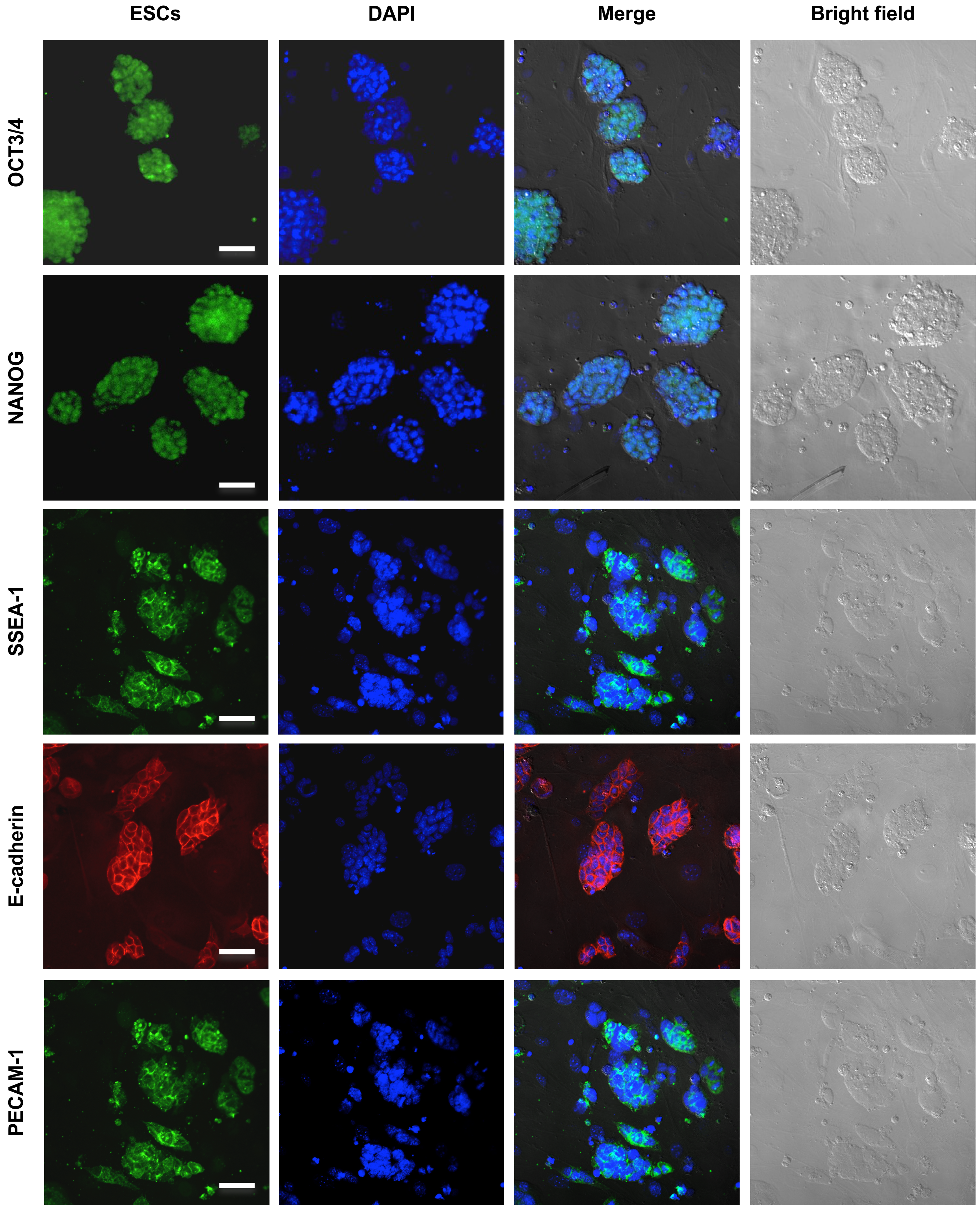

Supplement: S3 Fig — DAPI as used to stain DNA. A representative figure is shown. Scale bar, 50 μm. (TIF) [file pone.0130585.s004.tif]

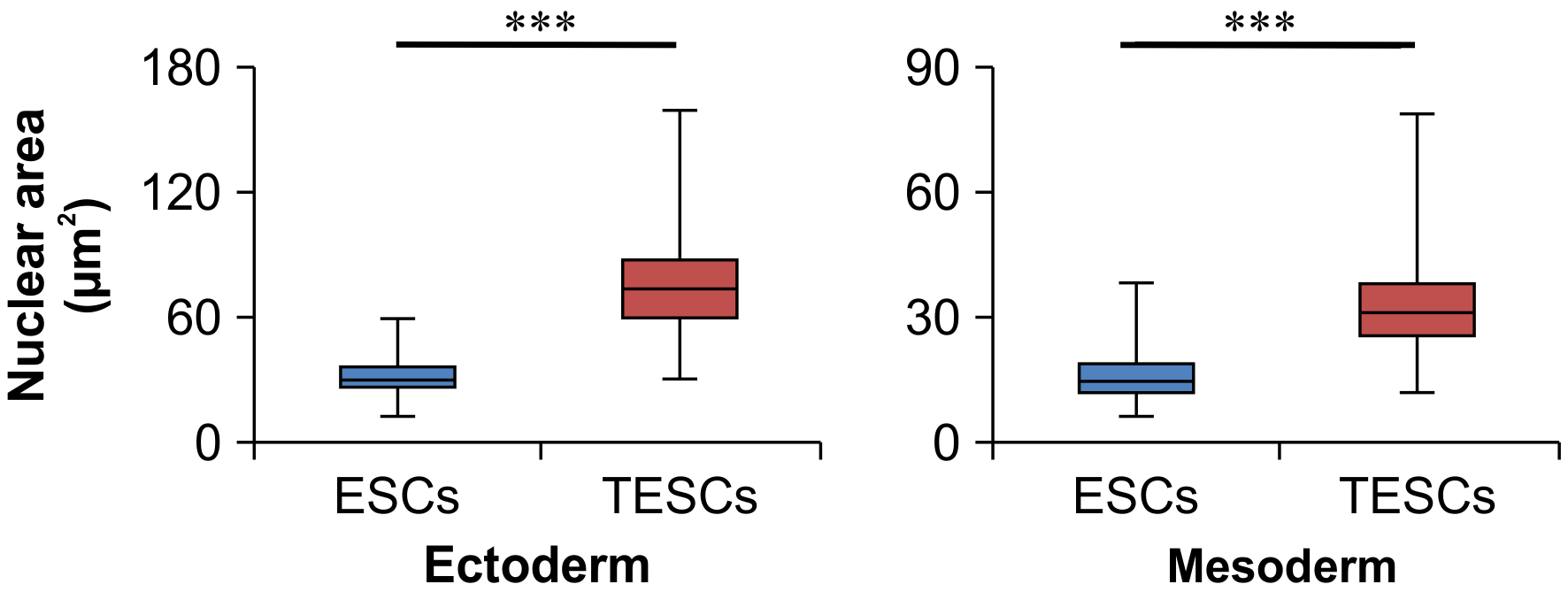

Supplement: S4 Fig — The nuclei of cells in ectodermal and mesodermal tissues within teratomas derived from TESCs were approximately twice as large as those from teratomas derived from ESCs. (TIF) [file pone.0130585.s005.tif]
