# Supplementary material for: Tetraploid Embryonic Stem Cells Maintain Pluripotency and Differentiation Potency into Three Germ Layers
Source: PLoS One. 2015 Jun 19;10(6):e0130585. doi: 10.1371/journal.pone.0130585 (PMC4474668; doi:10.1371/journal.pone.0130585)
Supplement: S1 Table — (DOCX) [file pone.0130585.s006.docx]

| Table S1. Primary antibodies | |  |
| --- | --- | --- |
| Primary antibody | Company | Catalog number |
| NANOG | Sigma | N3088 |
| OCT-4 | Sigma | SAB2701960 |
| SSEA-1 | Abcam | ab16285 |
| E-cadherin | Sigma | U3254 |
| PECAM-1 | Santa cruz | sc8306 |
