# Supplementary material for: Tetraploid Embryonic Stem Cells Maintain Pluripotency and Differentiation Potency into Three Germ Layers
Source: PLoS One. 2015 Jun 19;10(6):e0130585. doi: 10.1371/journal.pone.0130585 (PMC4474668; doi:10.1371/journal.pone.0130585)
Supplement: S2 Table — (DOCX) [file pone.0130585.s007.docx]

| Table S2. Efficiency of establishment of embryonic stem cells from diploid or tetraploid blastocysts | | | | |
| --- | --- | --- | --- | --- |
|  | Number of blastocyst outgrowths | Morphology (%) | | |
|  |  | Smooth rounded colonies | Syncytial giant cells | Spindle shaped cells |
|  |  |  |  |  |
| ESCs | 17 | 15 (88 %) | 14 (82 %) | 16 (94 %) |
| TESCs | 61 | 9 (15 %) | 50 (82 %) | 46 (75 %) |
| ESCs: embryonic stem cells from diploid blastocysts, TESCs: embryonic stem cells from tetraploid blastocysts | | | | |
