# Supplementary material for: Tetraploid Embryonic Stem Cells Maintain Pluripotency and Differentiation Potency into Three Germ Layers
Source: PLoS One. 2015 Jun 19;10(6):e0130585. doi: 10.1371/journal.pone.0130585 (PMC4474668; doi:10.1371/journal.pone.0130585)
Supplement: S3 Table — (DOCX) [file pone.0130585.s008.docx]

| Table S3. Differentiation of ESCs and TESCs in vivo | | |  |  |  |
| --- | --- | --- | --- | --- | --- |
| Cell type | Nude mice | | SCID mice | | Teratoma formation rate (%) |
|  | Number of s.c. mice | Teratoma formation | Number of s.c. mice | Teratoma formation |  |
| ESCs | 4 | 3 | 1 | 1 | 80 |
| TESCs | 4 | 0 | 3 | 1 | 14 |
